# Management of Colonic Diverticular Disease in the Older Adult

**DOI:** 10.1007/s11894-025-00986-4

**Published:** 2025-06-21

**Authors:** Hiep S. Phan, Lisa L. Strate

**Affiliations:** Division of Gastroenterology and Hepatology, School of Medicine and Public Health, University of Wisconsin, 1685 Highland Avenue, Madison, WI 53705 USA

**Keywords:** Diverticulosis, Diverticulitis, Diverticular bleeding, Older adult, Geriatric

## Abstract

**Purpose of Review:**

While societal guidelines help direct management of diverticulitis and diverticular bleeding broadly, our review focuses on the latest data for nuanced care of older patients affected by these conditions.

**Recent Findings:**

Diverticulitis in older patients can present with non-specific symptoms so a broad work up is recommended. Once diagnosed, those with uncomplicated disease (Hinchey Class 0 or 1a) can be safely managed without antibiotics or admission depending on frailty and comorbidities. Most older patients with complicated diverticulitis (abscess, perforation or obstruction) should be hospitalized. Elective or emergent surgery for complicated disease (Hinchey Class 1b–4) is associated with higher morbidity and mortality, particularly in older patients. The risk of diverticular bleeding and re-bleeding significantly increases with age, potentially due to the use of nonsteroidal anti-inflammatory drugs (NSAIDs) and anticoagulants.

**Summary:**

Diverticular disease and its associated complications disproportionately affect older adults. Management should focus on resuscitation, having low threshold for comprehensive work up and re-evaluating medication use for comorbid conditions to prevent recurrence.

## Introduction

Diverticular disease of the colon is one of the most common gastrointestinal disorders [1, 2]. Diverticulosis is a condition in which small saccular outpouchings form in the colon. Complications of diverticulosis, often termed diverticular disease, include diverticulitis (inflammation of one or a few adjacent diverticula and the surrounding colon and tissue) and diverticular bleeding (bleeding into the colon from a vessel in a single diverticulum). Diverticulitis is further categorized as complicated (diverticulitis with abscess, perforation, fistula and/or obstruction) or uncomplicated. Approximately 20–25% of individuals with incident diverticulitis will experience at least one recurrent episode [3]. Diverticular disease constitutes a significant burden on the healthcare system. In 2016, based on the National Ambulatory Medical Care Survey (NAMCS) and the National Hospital Ambulatory Medical Care Survey (NHAMCS), approximately one million patients were seen for diverticular-related diseases in the outpatient setting, and in 2018 according to the Nationwide Inpatient Sample approximately 325,000 patients were hospitalized for diverticulitis [2]. The financial burden related to the direct costs of care for diverticulitis is estimated to be $8.9 billion annually with approximately 80% of the costs resulting from inpatient hospital stays.

While management of diverticular disease has been outlined in societal guidelines, special considerations need to be taken with older patients who may have medical comorbidities and increased frailty. In this article, we will review the latest data on the diagnosis and medical management of diverticulitis and diverticular bleeding with a focus on older adults. Surgical management for diverticular disease is a complex issue regardless of age and will be briefly discussed in this review.

## Diverticulitis

### Epidemiology

Diverticulosis is the precursor lesion for diverticulitis. It affects up to 60% of individuals older than 60 years, and its prevalence increases to approximately 70% by age 85 years [1, 4]. The prevalence of diverticulosis is lower in patients younger than 50 years (25–35%) but has been steadily increasing in younger age groups for unclear reasons [5, 6]. 

**Fig. 1:**
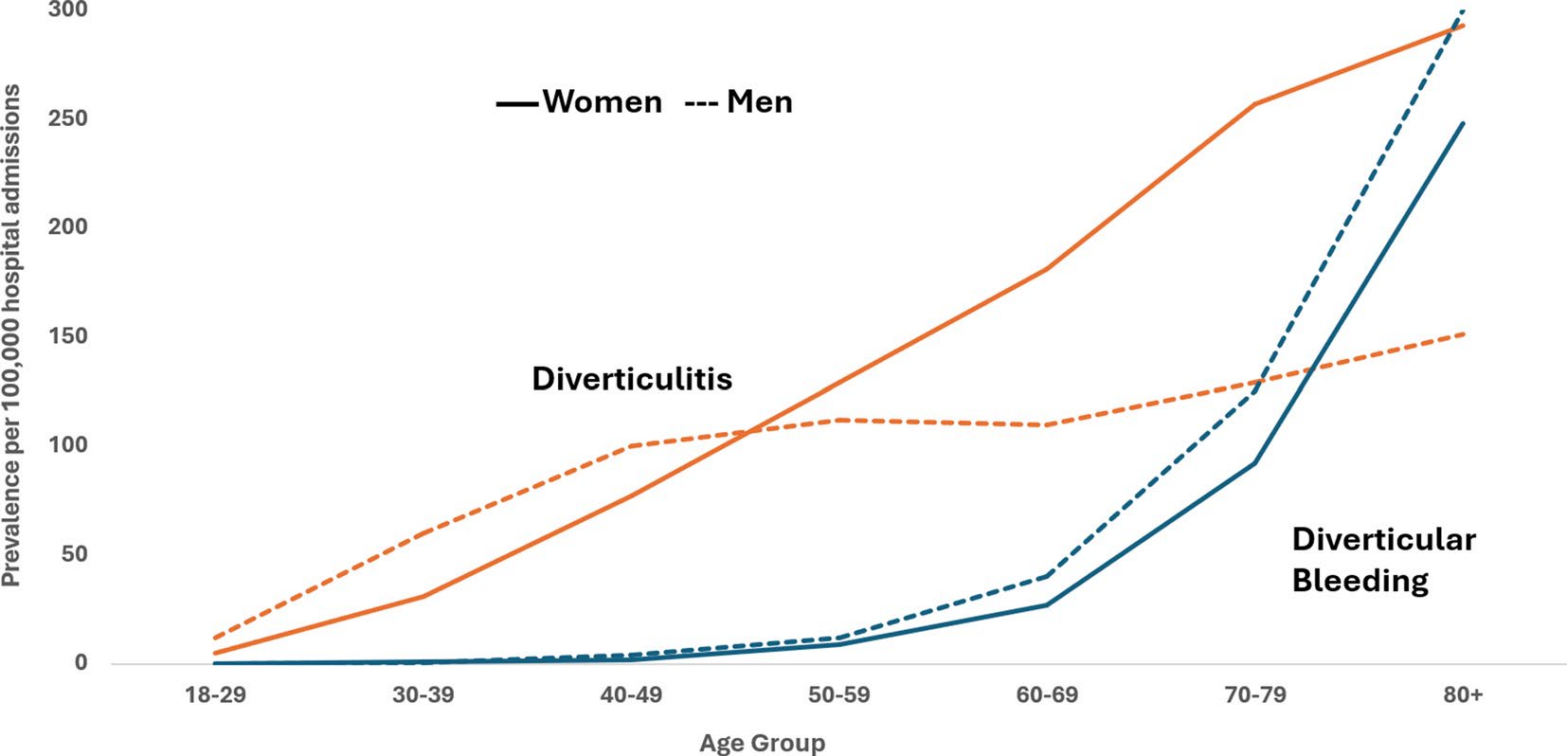
Prevalence of hospital admissions for diverticulitis and diverticular bleeding by sex and age in 2010

Approximately 2–4% of patients with diverticulosis will develop diverticulitis. The incidence of diverticulitis increases steadily with age (Fig. 1). Accordingly, the risk of developing diverticulitis increases based on time since diagnosis of diverticulosis, resulting in increased incidence with age (10 per 100,000 person-years in the second decade of life versus 535 per 100,000 in the ninth decade**).** [3, 7, 8] However, this also means that individuals who develop diverticulosis at a younger age have an increased risk of developing diverticulitis during their lifetime. For example, compared to the overall risk of 4%, individuals diagnosed with diverticulosis between ages 40 and 49 years had a risk of diverticulitis of 11% [7]. 

There is uncertainty regarding the impact of age on the severity of diverticulitis. A 20-year study of Olmstead County, Minnesota found that 63% of patients with complicated diverticulitis were aged 60 years or older compared to 7% in those younger than 40 years, and younger patients were at greater risk of recurrent diverticulitis [3]. However, a systematic review and meta-analysis of 27 studies found no differences in the rate of complications and need for emergency surgery in patients aged 50 years and older compared to younger patients [9]. This study also found no difference in recurrence rates when adjusted for duration of follow-up. These disparate results may be partially attributable to the age cut points used (60 vs. 50 years, respectively), and the fact that the meta-analysis included only studies of CT-confirmed diverticulitis.

The prevalence of diverticulitis in men versus women shifts dramatically with age. In younger individuals, diverticulitis is somewhat more common in men. However, starting in the 6th decade of life, it becomes more common in women and the gap between the prevalence in men and women widens with increasing age (Fig. 1). The reason for this epidemiological change is not known. However, one study found that menopausal hormone therapy was associated with increased risk, suggesting that sex hormones play a role [10]. 

### Presentation and Diagnosis

The initial presentation of diverticulitis generally includes non-specific symptoms such as constant abdominal pain exacerbated with movement, nausea without vomiting, diarrhea and/or constipation [11]. Typically, pain is focal in nature and located in the left-lower quadrant and/or supra-pubic area. However, interpreting presenting symptoms requires attention to patient demographics as there are variations associated with age and ethnicity. Abdominal pain can be the only presenting symptom for up to 20% of older patients [12]. In addition, patients of Asian descent are more likely to develop right-sided diverticulitis as opposed to left -sided predominance in western society, which can be misdiagnosed as appendicitis [13]. Therefore, work up requires considering a broad differential diagnosis (Table 1). Diagnoses that are common in older adults presenting with acute or subacute abdominal pain include ischemic colitis, colonic volvulus, inflammatory bowel disease and colorectal cancer. Pain associated with ischemic colitis and inflammatory bowel disease is generally more diffuse and diarrhea is a prominent symptom. Abdominal distention and obstipation are hallmarks of a volvulus. Pain is a less common manifestation of colon cancer and symptoms usually have a gradual onset.

**Table 1:** Differential diagnosis and Work-up for acute diverticulitis in older adults

Diagnosis	Classic Clinical History	Clinical Pearls in Older Adults
Colorectal Cancer	Progressive constipation with changes over weeks to months Abdominal pain usually due to advanced disease	Review of family history and prior colorectal cancer screening helps determine pre-test probability Possible lab findings include elevated carcinoembryonic antigen (CEA) Local bowel wall thickening without inflammation
Inflammatory Bowel Disease (IBD)	Subacute to chronic abdominal pain, diarrhea, and hematochezia Constitutional symptoms including weight loss, fever, and fatigue	~ 10–15% of IBD is initially diagnosed in patients > 60 years old [48] Constitutional symptoms tend the be less prevalent in older adults [49] Reliant on endoscopic and histologic confirmation due to symptom overlap with diverticulitis
Ischemic Colitis	Can be acute (within 24 h) or less commonly chronic (loose definition of weeks to months after acute) [50] Pain develops prior to diarrhea or hematochezia Associated with cardiovascular comorbidities, IBS and constipation.	Chronic ischemia occurs in up to 18% without clear risk factors [51] Elevated lactate, lactate dehydrogenase or white blood cell is associated with advance disease
Infectious Colitis	Typically, inciting event within 1–3 days (incubation period) prior to symptoms Generalized abdominal pain Watery/profuse diarrhea	Review of recent antibiotic exposure and travel is helpful to determine potential source Stool testing with stool pathogen PCR or culture Usually, continuous bowel wall thickening and inflammation on imaging [52]
Symptomatic diverticular disease	Chronic, intermittent pain in the left lower quadrant Intermittent bowel habit changes	Difficult to distinguish from irritable bowel syndrome Normal labs, imaging and endoscopy
Irritable Bowel Syndrome (IBS)	Chronic abdominal pain with diarrhea/constipation Younger age at onset Female predominance	Prevalence decreases with age and lower likelihood for initial diagnosis in older adults [47] Diagnosis of exclusion with normal labs and imaging and Rome IV Criteria
Bowel Obstruction/Volvulus	Constipation/Obstipation over a few days with worsening abdominal pain Advanced disease with nausea and vomiting	~ 80% of patients with prior volvulus that is not surgically treated have recurrence [53] Prior abdominal surgeries or IBD increase likelihood Dilated bowel with transition point on imaging – typically sigmoid in older patients
Acute Appendicitis	Acute periumbilical to right lower quadrant pain Younger age	Appendiceal abnormality on imaging Higher risk of appendiceal tumor in complicated appendicitis in older patients [54]
Biliary disease (choledocholithiasis, cholecystitis)	Acute epigastric/right upper quadrant pain; radiates to back; association with eating Jaundice	Can have elevated liver enzymes Gallbladder wall thickening or dilated bile ducts on ultrasound or CT scan Elevated CA 19 − 9 can be elevated during acute inflammation; need to recheck after resolution [55]
Gynecologic Diseases (Uterine fibroids, pregnancy, infections)	Subacute to chronic abdominal pain without bowel habit changes Irregular menstruation Vaginal discharge +/- adnexal/cervical motion tenderness	Pregnancy testing depending on age and reports of menstruation; rarely needed in older adults Gynecologic abnormality on imaging Positive sexually transmitted disease testing
Urinary Tract Diseases (Urinary tract infection, pyelonephritis, nephrolithiasis)	Acute back/flank/suprapubic pain Hematuria Dysuria	Older adults can present with minimal urinary symptoms [56] Minimal evidence to suggest falls or encephalopathy as sole symptoms for urinary tract disease [57, 58] Urinalysis – Leukocyte esterase, nitrite, sediments

The classic presentation of diverticulitis consists of leukocytosis, elevated inflammatory markers, fever and abdominal pain. Predictors of diverticulitis in patients presenting with abdominal pain include age older than 50 years, as well as a prior diagnosis of diverticulitis, pain and tenderness localized to the left lower quadrant, aggravation of pain with movement and absence of vomiting [14]. However, not all patients manifest these signs and symptoms, particularly older adults, and prediction rules based on these factors alone have not performed optimally [14]. In addition, complications of diverticulitis including abscess, perforation, obstruction and fistula, which require more aggressive treatment algorithms, are not always evident on history and physical exam. Accordingly, cross-sectional imaging with abdominal pelvic computed tomography (CT) scanning has been widely adopted and recommended by societal guidelines as an integral component of the clinical toolkit [15–20]. 

Studies indicate that the clinical presentation in older patients is more nuanced than in younger individuals. Older adults are less likely to have leukocytosis and fever, and more likely to present with atypical symptoms such as fatigue and rectal bleeding [21, 22]. In one study of 464 patients older than 80 years presenting to the emergency department with abdominal pain, only 67% were suspected to have diverticulitis prior to imaging [23]. Therefore, guidelines recommend a CT with IV contrast in all patients older than age 65 years with suspected diverticulitis. Measures can be taken to reduce IV contrast complications in patients with chronic kidney disease and other risk factors such as avoiding multiple closely spaced exposures to contrast and minimizing contrast volume. Alternatively, CT-scan without contrast or MRI can be used for the diagnosis [24]. Point of care ultrasound is a diagnostic alternative but is operator dependent, may miss complications including free air, and is not widely used in the United States [25]. Occasionally, a CT scan may not be necessary. For example, patients with a history of CT-confirmed diverticulitis who present with stereotypical and mild symptoms can be diagnosed without CT. High values of C-reactive protein (CRP) are indicative of complications and should prompt a CT scan.

### Management

The management of diverticulitis depends on the presence or absence of complications, the severity of the clinical presentation (e.g. signs and symptoms of sepsis, peritonitis), and the burden of comorbid disease. The Modified Hinchey Classification is a CT scan-based system developed to guide surgical management and remains the basis of many treatment guidelines. It consists of four stages ranging from peri diverticular colon wall thickening alone(stage 0), wall thickening with reaction in the peri-colonic fat (stage 1a), small (< 3–4 cm) peri-colonic or mesenteric abscess (stage 1b), intra-abdominal abscess distant from the primary inflammatory process or in the pelvis or retroperitoneum (stage II), generalized purulent peritonitis (stage III), and generalized fecal peritonitis (stage IV).

Patients with Hinchey Class 0 and Ia are considered to have uncomplicated diverticulitis. The recommendation for most patients with uncomplicated diverticulitis is conservative management with several days of a liquid or low-residue diet, acetaminophen and antispasmodics for pain control. Four well-designed randomized controlled trials in Europe and Oceania have shown that antibiotics impart no benefit on time to symptom resolution, risk of short and long-term complications, recurrence and mortality. Three of the four trials did not exclude patients based on age and one included adults younger than 80 years of age. The average age of trial participants ranged from 56 to 59 years. These results suggest that a conservative approach without antibiotics is safe in older patients with uncomplicated diverticulitis if they do not have signs of sepsis or peritonitis or risk factors for progression to complicated disease or failure of conservative management [26–29]. Such factors include immunocompromise, high burden of comorbid disease, lack of follow up, frailty, and severe presentation (e.g. systemic inflammatory response syndrome) [15, 31]. These factors are prevalent in older adults and are associated with adverse outcomes. In an administrative database study of patients older than 75 years, those who were frail (Hospital Frailty Risk Score ≥ 5) were more likely to die, experience complications, undergo more invasive surgeries and to stay in the hospital longer [32]. Therefore, treatment decisions for older adults with uncomplicated diverticulitis must consider comorbid illness, frailty and social support.

If antibiotics are needed in the outpatient setting, a fluroquinolone with metronidazole or amoxicillin-clavulanate alone are recommended. There are no randomized trials comparing various antimicrobial agents for diverticulitis. However, a study using a nationwide claims database and Medicare data (individuals aged 65 years or older) compared these two antibiotic regimens in the outpatient setting. Although there were no differences in diverticulitis outcomes, the 1-year risk of *Clostridioides difficile* infection (CDI) was higher for the fluroquinolone plus metronidazole group than for the amoxicillin- clavulanate group in the Medicare Cohort [33]. In addition, older patients are at increased risk of tendon injury while on fluoroquinolones, and the risk increases with duration of treatment [34]. Therefore, special attention should be paid to the selection of antibiotics based on the risk of complications in older patients [33]. The duration of therapy is another area of uncertainty. One study of patients with uncomplicated diverticulitis found that a 4-day course resulted in similar outcomes to 7 days [35]. Therefore, it is reasonable to treat with 4 days and extend for persistent symptoms. A shorter course is also likely to minimize antibiotic side effects.

In general, immunocompetent and otherwise healthy patients with uncomplicated diverticulitis (Hinchey Class 0 to Ia) who have adequate cognitive capacity and support systems can be managed in the outpatient setting regardless of treatment with antibiotics. A noninferiority trial of 480 patients aged 18 to 80 years found no differences in the need for hospital admission, number of emergency department visits, pain control and emergency surgery between patients managed with and without antibiotics in the outpatient setting [28]. However in observational studies, increasing age after adjustment for comorbidity burden is associated with a higher likelihood of hospital admission after presenting to the emergency department [36]. Therefore, close follow-up is recommended for older patients treated in the outpatient setting.

Management of diverticulitis complicated by an abscess (particularly those large than 3 cm) or perforation (Hinchey Class Ib to IV) usually requires admission for intravenous antibiotics with potential percutaneous drainage and/or surgical intervention, and older patients with an abscess should be admitted. The management of complicated diverticulitis is nuanced and most often falls in the purview of surgical services, which is outside of the scope of this review. However, increasing patient age after accounting for comorbidity is a risk factor for surgical intervention (45% increased odds comparing patients aged 65 years and older to those less than 45 years) and in-hospital mortality (6 times increased odds comparing those 65 years and older to less than 45 years) [36]. In addition, older age is a predictor of postoperative mortality following emergency surgery for diverticulitis particularly in patients who have any complication or require reoperation. Patients aged 65–79 years with postoperative complications have a 4-fold risk of mortality, and those older than 80 years have a 10-fold greater risk compared to those younger than 65 years after adjustment for comorbid disease and other perioperative characteristics [37]. Another study found that mortality dramatically increased with advancing age after both emergent and elective surgery for diverticulitis. However, the difference in mortality between young and older patients was the most pronounced in the elective setting [38]. These studies indicate the importance of pre-operative risk assessment and optimization and care in the perioperative period in older patients.

### Post-acute Management

Traditionally, guidelines recommended colonoscopy following a diverticulitis episode based on studies of patients undergoing colonoscopy finding increased detection of cancers and advanced adenomas compared to estimates from studies of routine screening [39]. In addition, cohort studies found the risk of colorectal cancer was higher in individuals with a history of diverticulitis, particularly in the first year of follow-up [40]. However, an updated meta-analysis of 31 studies found that the pooled prevalence of colorectal cancer after diverticulitis was 1.9%. The risk was substantially higher after an episode of complicated diverticulitis (7.9%) than uncomplicated diverticulitis (1.3%). Meta-regression did not find any association between age and risk of colorectal cancer in patients after diverticulitis [41]. Based on this study and another meta-analysis, recent guidelines suggest physicians refer patients for colonoscopy after an initial episode of complicated diverticulitis if they have not had a recent colonoscopy. Patients with uncomplicated diverticulitis should be referred for colonoscopy if they are not up-to-date with colorectal cancer screening or they have worrisome symptoms (such as rectal bleeding and progressive constipation) or findings on imaging suspicious for colon malignancy [42]. 

Approximately 8% of patients with incident diverticulitis will experience a recurrence within the first year and 22% by 10 years of follow-up. The risk of recurrence increases further with each subsequent diverticulitis episode [3]. As noted previously, it is unclear how age impacts risk of recurrence. Nonetheless, prevention is important. Some risk factors for recurrence such as severity of incident disease and family history of diverticulitis cannot be modified [43]. However, diet and lifestyle factors have been associated with decreased risk of incident diverticulitis, and therefore likely alter risk of recurrent disease. These include eating a diet high in fiber and low in red meat, engaging in regular physical activity, maintaining a healthy body weight, and avoiding smoking and heavy alcohol consumption. A prospective study of over 50,000 men found that adherence to 5 low-risk diet and lifestyle factors was associated with a 75% decreased risk of incident diverticulitis [44]. Avoiding nonsteroidal anti-inflammatory drugs and opiates may also decrease risk particularly of complicated diverticulitis [45]. Recommendations regarding prevention are the same regardless of age and adhering to these healthy practices may decrease the risk of other chronic diseases as well.

A poorly defined proportion of patients who have had diverticulitis will develop chronic symptoms. Some patients (< 10%) have refractory or smoldering diverticulitis defined as diverticulitis that fails to resolve with treatment or that returns shortly afterwards. These patients require long courses of antibiotics which as noted above can put older patients at risk of side effects. Those who do not respond often require segmental colectomy [25]. It is also possible for patients to develop visceral hypersensitivity following diverticulitis similar to post-infectious irritable bowel syndrome. Compared to patients with smoldering diverticulitis, these patients will have a normal CT scan, laboratory tests and endoscopy and do not respond to antibiotics. In addition, symptoms will be more protracted without symptom-free periods between episodes. Symptomatic uncomplicated diverticular disease is a term used to describe symptoms in individuals with diverticulosis who do not have evident diverticulitis. It is controversial whether diverticulosis without prior diverticulitis results in chronic symptoms, but both diverticulosis and irritable bowel syndrome are very common and can co-occur [46, 47]. Nonetheless, new symptoms in an older adult should not be assumed to be due to irritable bowel syndrome and deserve further attention.

## Diverticular Bleeding

### Epidemiology

Based on nationwide data, there were approximately 1.7 million outpatient visits for gastrointestinal (GI) bleeding in the US in 2018 [2]. Of those, approximately 460,000 visits were related to a lower GI source. It is estimated that diverticular bleeding accounts for 27–64% of lower GI bleeding cases [58–61]. Overall, diverticular bleeding is less common than diverticulitis. In one study, the prevalence of admission for diverticular bleeding was 27 per 100, 000 hospitalizations compared to 92 per 100,000 for diverticulitis [8]. However, risk of developing incident diverticular bleeding increases by more than 200 fold between the 3rd and 9th decades of life [63, 64], so that by the 9th decade diverticular bleeding is twice as common as diverticulitis **(**Fig. 1**)** [8]. Furthermore, older patients with lower GI bleeding are at the highest risk for prolonged hospitalization and mortality [64].

### Presentation and Diagnosis

Diverticular bleeding is heralded by painless, large volume hematochezia (maroon to red blood depending on location and rate). Generally accepted signs of severe bleeding include shock (hypotension, tachycardia, and syncope) and/or transfusion requirement [65, 66]. Patients older than 75-years-old have a 4-fold increased risk for developing a severe bleeding [66], and older age is a predictor of poor outcome, including mortality [67]. Other risk factors for severity include non-steroidal anti-inflammatory drug (NSAID) use and high burden of comorbid disease, which are common in older adults [66–68]. Therefore, prompt evaluation and resuscitation is crucial in older patients.

Once the patient has been resuscitated and stabilized, work up should focus on identifying the source of bleeding. In addition to diverticular bleeding, other bleeding etiologies that are more common in older adults include angiodysplasia, ischemic colitis, and colorectal cancer. Angiodysplasia usually manifests as obscure blood loss; ischemic colitis as diarrhea and abdominal pain, and colorectal cancer as chronic bleeding with iron deficiency anemia and change in stool habits (Table 2).

**Table 2:** Differential diagnosis and Work-up for diverticular bleeding in older adults

Diagnosis	Classic Clinical History	Clinical Pearls in Older Adults
Post-polypectomy	Acute bleeding within 3 weeks of colonoscopy Higher risk with hot snare for delayed bleeding vs. cold snare	Right colon site, polyp size > 10 mm, pedunculated polyp are risk factors; age is not a risk factor [95] Evaluate with CT angiography (CTA) if brisk Mange with repeat colonoscopy if ongoing with supportive management
Ischemic colitis	Can be acute (within 24 h) or less commonly chronic (loose definition of weeks to months after acute) [50] Pain development prior to diarrhea or hematochezia Associated with cardiovascular comorbidities, IBS and constipation.	Chronic ischemia prevalence estimated to be up to 18% without clear risk factors [51] Elevated lactate, lactate dehydrogenase or white blood cell is associated with advance disease
Hemorrhoids	Acute, intermittent hematochezia “Rush” of blood in the toilet Usually painless; can be painful with thrombosed or external hemorrhoids	Peak prevalence usually in 40–65 years old patients with decrease prevalence after 65 [96] Blood on rectal exam – internal hemorrhoids rarely palpated or seen without anoscopy or colonoscopy
Stercoral ulcer	Common symptoms include abdominal pain, distension, nausea before hematochezia Associated with chronic constipation	Older patients and those with cognitive impairment can present with vague symptoms (fatigue, weakness, encephalopathy) [97] Common findings of large stool burden on CT scan +/- associated colitis Disease severity can be assessed with flexible sigmoidoscopy or colonoscopy following bowel purge
Angiodysplasia	Intermittent painless bleeding Often presents as occult blood loss presenting with symptoms of anemia	Risk factors include older age (> 50 years old) [98], chronic renal disease [99], aortic stenosis and von Willebrand disease [100] Colonoscopy with treatment of both bleeding & nonbleeding lesions CTA if brisk bleeding
Inflammatory bowel disease	Subacute to chronic abdominal pain, diarrhea, and hematochezia Constitutional symptoms with weight loss, fever, and fatigue	~ 10–15% of IBD is initially diagnosed in patients > 60 years old [48] Constitutional symptoms tend the be less prevalent in older adults[49] Reliant on endoscopic and histologic confirmation due to symptom overlap with diverticulitis
Colorectal cancer	Progressive constipation with changes over weeks to months Abdominal pain usually due to advanced disease	Review of family history and prior colorectal cancer screening helps determine pre-test probability Possible lab findings include elevated carcinoembryonic antigen (CEA) Local bowel wall thickening without inflammation

CT angiography (CTA) is a readily available, noninvasive diagnostic test for GI bleeding that is recommended as the initial test in patients with hemodynamically significant bleeding. [70] However, it identifies bleeding in approximately 15% of patients with diverticular bleeding [71, 72], because CTA relies on active bleeding for a positive result and most diverticular bleeding resolves spontaneously or is intermittent [68, 73]. A definitive endoscopic diagnosis is also difficult given that stigmata of diverticular bleeding are elusive. In a systemic review of 11 studies, only 16% of cases underwent endoscopic intervention [74]. Randomized trials have shown no diagnostic or therapeutic benefit to urgent vs. nonemergent colonoscopy [75, 76]. Furthermore, several studies have indicated that urgent endoscopy may increase the risk of poor outcome in part due to insufficient resuscitation [77]. Accordingly, guidelines recommend non-emergent colonoscopy after adequate resuscitation for most patients with lower GI bleeding. [70] In patients with self-limited bleeding and a recent high-quality colonoscopy finding diverticulosis, additional work up may not be necessary. [70] The risks and benefits of endoscopic evaluation should be carefully considered in older patients with comorbid disease and/or frailty. For example, in an older, frail patient with self-limited bleeding and no findings concerning for malignancy, colonoscopy may be deferred.

### Acute Management

Initial management should involve assessment of bleeding severity and resuscitation. There are various published prediction tools available to aid in triage. However, these are best for identifying low-risk patients and do not accurately predict those in need of therapeutic interventions [78]. Since age and comorbid disease are predictors of poor outcome in most models, most older patients with acute hematochezia should be hospitalized. Transfusion thresholds for lower GI bleeding have been extrapolated from upper GI bleeding studies in which a restrictive hemoglobin threshold (> 7 g/dl) improved mortality compared to a threshold of > 9 g/dl [79]. However, the decision to transfusion must be individualized particularly in older patients [80]. Rapid blood loss and conditions that reduce tolerance to lower hemoglobin levels should push the transfusion threshold upwards, whereas a threshold of 7 g/dl may be appropriate in patients with modest blood loss and few comorbidities or in those where hypervolemia is a concern. Platelet transfusion is indicated in patients with severe bleeding and platelet count < 30 K; in patients on aspirin and/or other antiplatelet agents without thrombocytopenia platelet transfusion may increase mortality [70, 81].

Many older patients have cardiovascular or venous thromboembolic (VTE) disease requiring antiplatelet and/or anticoagulant medications that increase risk for severe bleeding [67]. A multidisciplinary approach is recommended for these patients particularly those with recently placed cardiac stents or at very high risk of thromboembolic events. Aspirin used for secondary prevention should be continued in most patients, and non-aspirin antiplatelets held temporarily in patients with severe bleeding [82]. The decision to reverse anticoagulation is based on the indication, degree of anticoagulation and severity of bleeding [70]. Colonoscopy with endoscopic treatment is considered safe in the setting of INR ≤ 2.5 [83]. For warfarin, the use of prothrombin complex concentrate (PCC) is favored over fresh frozen plasma (FFP) due to rapid correction and lower risk for volume overload [84, 85]. Targeted reversal agents are available for some Direct Oral Anticoagulants (DOACs; idarucizumab for dabigatran; andexanet al.fa for apixaban and rivaroxaban). However, these should only be used in patients with life-threatening bleeding that does not respond to resuscitation. The use of anti-fibrinolytics such as tranexamic acid is not recommended as it does not improve transfusion requirements or hospitalization length and increases risk of VTE events [86, 87]. 

For patients who have a source of bleeding identified on CTA, prompt transcatheter angiography with embolization of a bleeding source is recommended. The longer the delay between a positive CTA and angiography, the lower the likelihood of finding active bleeding and performing embolization [88]. Renal toxicity should be mitigated as possible in older adults undergoing CTA and angiography. Nonurgent colonoscopy is the next step for most stable patients with a negative CTA. When detected, stigmata of recent hemorrhage should be treated endoscopically, as this reduces risk for early and late recurrent bleeding [89]. Mechanical methods such as through- the-scope clips and band ligation are recommended over thermal methods for diverticular stigmata due to the potential risk of perforation. There are no data suggesting that the effectiveness of endoscopy treatment of diverticular stigmata differs in older patients.

### Re-bleeding Risk

The risk of recurrent diverticular bleeding increases with age. There is a 2-fold increase between patients in the 5th decade of life compared to those in the 8th decade [90]. NSAID and anticoagulation use also increase risk [91], whereas, NSAID cessation after an initial episode has been shown to decrease recurrence (9.4% vs. 77%) [92, 93]. However, cessation of aspirin for secondary prevention is associated with increase all-cause mortality and should be continued in most patients [82]. A small retrospective study found that patients on DOACs required fewer transfusions and experienced fewer recurrent episodes when compared to those on warfarin [94]. 

## Conclusion

As the aging population continues to grow worldwide, diverticular disease and its associated complications will become more prevalent. The management of diverticulitis and diverticular bleeding in older adults requires special attention. The thresholds for a diagnostic CT scan, antibiotic treatment and hospitalization are lower in older adults with diverticulitis particularly those with comorbid conditions. Older adults, particularly those who undergo surgery for diverticulitis, are at increased risk of morbidity and mortality so careful decision making and perioperative care are crucial. Older patients with diverticular bleeding are more likely to experience severe bleeding in part due to the prevalence of risk factors in this age group including anticoagulant and antiplatelet use. Therefore, resuscitation is important, and management decisions must consider risks and benefits.

## Key References

- Qaseem A, Etxeandia-Ikobaltzeta I, Lin JS, Fitterman N, Shamliyan T, Wilt TJ. Diagnosis and Management of Acute Left-Sided Colonic Diverticulitis: A Clinical Guideline From the American College of Physicians. Annals of Internal Medicine. 2022;175 (3):399–415. 10.7326/m21-2710.
-
  - Recent guideline from the American College of Physicians covering care of patients presenting with diverticulitis. It also includes a concise review of the evidence.
- Sengupta N, Feuerstein JD, Jairath V, Shergill AK, Strate LL, Wong RJ, et al. Management of Patients With Acute Lower Gastrointestinal Bleeding: An Updated ACG Guideline. Am J Gastroenterol. 2023;118 (2):208–31.
-
  - A recent guideline on the management of lower gastrointestinal bleeding from the American College of Gastroenterology that includes a comprehensive review of the literaure as well as sections specific to diverticular bleeding.
- Zhao Y, Gao Y, Chen W, Hao Q, Ho C, Kennedy MK, et al. Antibiotics for treatment of mild left-sided acute uncomplicated diverticulitis: meta-analysis of randomized trials. Br J Surg. 2023;110 (3):373–4.
-
  - A comprehensive meta-analysis including data from all randomized trials to date comparing antibiotic versus no antibiotics for the treatment of uncomplicated left-sided diverticulitis.

## Data Availability

No datasets were generated or analysed during the current study.

## References

[1] Peery AF, Keku TO, Martin CF, Eluri S, Runge T, Galanko JA, et al. Distribution and characteristics of colonic diverticula in a united States screening population. Clin Gastroenterol Hepatol. 2016;14(7):980–e51. 26872402 10.1016/j.cgh.2016.01.020 PMC4912930

[2] Peery AF, Crockett SD, Murphy CC, Jensen ET, Kim HP, Egberg MD, et al. Burden and cost of gastrointestinal, liver, and pancreatic diseases in the united States: update 2021. Gastroenterology. 2022;162(2):621–44. 34678215 10.1053/j.gastro.2021.10.017 PMC10756322

[3] Bharucha AE, Parthasarathy G, Ditah I, Fletcher JG, Ewelukwa O, Pendlimari R, et al. Temporal trends in the incidence and natural history of diverticulitis: A Population-Based study. Am J Gastroenterol. 2015;110(11):1589–96. 26416187 10.1038/ajg.2015.302 PMC4676761

[4] Everhart JE, Ruhl CE. Burden of digestive diseases in the united States part II: lower Gastrointestinal diseases. Gastroenterology. 2009;136(3):741–54. 19166855 10.1053/j.gastro.2009.01.015

[5] Wlodarczyk JR, Yoon D, Owens J, Ershadi S, Lee SW, Cologne KG, et al. Prevalence of and risk factors for incidental colonic diverticulosis. J Surg Res. 2022;280:348–54. 36037611 10.1016/j.jss.2022.07.021

[6] De Cecco CN, Ciolina M, Annibale B, Rengo M, Bellini D, Muscogiuri G, et al. Prevalence and distribution of colonic diverticula assessed with CT colonography (CTC). Eur Radiol. 2016;26(3):639–45. 26105021 10.1007/s00330-015-3866-1

[7] Shahedi K, Fuller G, Bolus R, Cohen E, Vu M, Shah R, et al. Long-term risk of acute diverticulitis among patients with incidental diverticulosis found during colonoscopy. Clin Gastroenterol Hepatol. 2013;11(12):1609–13. 23856358 10.1016/j.cgh.2013.06.020 PMC5731451

[8] Wheat CL, Strate LL. Trends in hospitalization for diverticulitis and diverticular bleeding in the united States from 2000 to 2010. Clin Gastroenterol Hepatol. 2016;14(1):96–103. e1. 25862988 10.1016/j.cgh.2015.03.030 PMC4624035

[9] van Dijk ST, Abdulrahman N, Draaisma WA, van Enst WA, Puylaert JBCM, de Boer MGJ, et al. A systematic review and meta-analysis of disease severity and risk of recurrence in young versus elderly patients with left-sided acute diverticulitis. Eur J Gastroenterol Hepatol. 2020;32(5):547–54. 31972659 10.1097/MEG.0000000000001671

[10] Jovani M, Ma W, Joshi AD, Liu PH, Nguyen LH, Cao Y, et al. Menopausal hormone therapy and risk of diverticulitis. Am J Gastroenterol. 2019;114(2):315–21. 30730324 10.14309/ajg.0000000000000054 PMC8074020

[11] Laurell H, Hansson LE, Gunnarsson U. Acute diverticulitis–clinical presentation and differential diagnostics. Colorectal Dis. 2007;9(6):496–501. discussion– 2. 17573742 10.1111/j.1463-1318.2006.01162.x

[12] Lee TH, Setty PT, Parthasarathy G, Bailey KR, Wood-Wentz CM, Fletcher JG et al. Aging, Obesity, and the Incidence of Diverticulitis: A Population-Based Study. Mayo Clin Proc. 2018;93(9):1256–65. 10.1016/j.mayocp.2018.03.005 PMC6200415 30193674

[13] Uhe I, Meyer J, Viviano M, Naiken S, Toso C, Ris F, et al. Caecal diverticulitis can be misdiagnosed as acute appendicitis: a systematic review of the literature. Colorectal Dis. 2021;23(10):2515–26. 34272795 10.1111/codi.15818 PMC9292704

[14] Kiewiet JJ, Andeweg CS, Laurell H, Daniels L, Lameris W, Reitsma JB, et al. External validation of two tools for the clinical diagnosis of acute diverticulitis without imaging. Dig Liver Dis. 2014;46(2):119–24. 24252579 10.1016/j.dld.2013.09.017

[15] Peery AF, Shaukat A, Strate LL. AGA clinical practice update on medical management of colonic diverticulitis. Expert Rev Gastroenterol. 2021;160(3):906–11. e1. 10.1053/j.gastro.2020.09.059 PMC7878331 33279517

[16] Qaseem A, Etxeandia-Ikobaltzeta I, Lin JS, Fitterman N, Shamliyan T, Wilt TJ. Diagnosis and management of acute Left-Sided colonic diverticulitis: A clinical guideline from the American college of physicians. Ann Intern Med. 2022;175(3):399–415. 35038273 10.7326/M21-2710

[17] Hall J, Hardiman K, Lee S, Lightner A, Stocchi L, Paquette IM, et al. The American society of Colon and rectal surgeons clinical practice guidelines for the treatment of Left-Sided colonic diverticulitis. Dis Colon Rectum. 2020;63(6):728–47. 32384404 10.1097/DCR.0000000000001679

[18] Schultz JK, Azhar N, Binda GA, Barbara G, Biondo S, Boermeester MA, et al. European society of coloproctology: guidelines for the management of diverticular disease of the colon. Colorectal Dis. 2020;22(Suppl 2):5–28. 32638537 10.1111/codi.15140

[19] Nagata N, Ishii N, Manabe N, Tomizawa K, Urita Y, Funabiki T, et al. Guidelines for colonic diverticular bleeding and colonic diverticulitis: Japan gastroenterological association. Digestion. 2019;99(Suppl 1):1–26. 30625484 10.1159/000495282

[20] Solomkin JS, Mazuski JE, Bradley JS, Rodvold KA, Goldstein EJ, Baron EJ, et al. Diagnosis and management of complicated intra-abdominal infection in adults and children: guidelines by the surgical infection society and the infectious diseases society of America. Surg Infect (Larchmt). 2010;11(1):79–109. 20163262 10.1089/sur.2009.9930

[21] Covino M, Rosa F, Ojetti V, Quero G, Fiorillo C, Sganga G, et al. Acute diverticulitis in elderly patients: does age really matter?? Dig Dis. 2021;39(1):33–41. 32485716 10.1159/000509049

[22] Sasaki Y, Komatsu F, Kashima N, Maeda T, Urita Y. Reactive leukocytosis in older patients with acute colonic diverticulitis: A retrospective study utilizing logistic regression analysis. Geriatr Gerontol Int. 2020;20(10):951–5. 32876981 10.1111/ggi.14027 PMC7590047

[23] Gardner CS, Jaffe TA, Nelson RC. Impact of CT in elderly patients presenting to the emergency department with acute abdominal pain. Abdom Imaging. 2015;40(7):2877–82. 25862547 10.1007/s00261-015-0419-7

[24] Fugazzola P, Ceresoli M, Coccolini F, Gabrielli F, Puzziello A, Monzani F, et al. The WSES/SICG/ACOI/SICUT/AcEMC/SIFIPAC guidelines for diagnosis and treatment of acute left colonic diverticulitis in the elderly. World J Emerg Surg. 2022;17(1):5. 35063008 10.1186/s13017-022-00408-0 PMC8781436

[25] Morris AM, Fider JL, Mau B, Strate LL. Recurrent lower abdominal pain, altered bowel habits, and malaise: Conservative or surgical approach to a common disorder. Gastroenterology. 2023;164(4):541–e91. 36657527 10.1053/j.gastro.2023.01.011

[26] Chabok A, Pahlman L, Hjern F, Haapaniemi S, Smedh K, Group AS. Randomized clinical trial of antibiotics in acute uncomplicated diverticulitis. Br J Surg. 2012;99(4):532–9. 22290281 10.1002/bjs.8688

[27] Daniels L, Unlu C, de Korte N, van Dieren S, Stockmann HB, Vrouenraets BC, et al. Randomized clinical trial of observational versus antibiotic treatment for a first episode of CT-proven uncomplicated acute diverticulitis. Br J Surg. 2017;104(1):52–61. 27686365 10.1002/bjs.10309

[28] Mora-Lopez L, Ruiz-Edo N, Estrada-Ferrer O, Pinana-Campon ML, Labro-Ciurans M, Escuder-Perez J Efficacy and Safety of Nonantibiotic Outpatient Treatment in Mild Acute Diverticulitis (DINAMO-study): A Multicentre, Randomised, Open-label, Noninferiority Trial. Ann Surg. 2021;274(5):3435–e42 10.1097/SLA.0000000000005031 34183510

[29] Zhao Y, Gao Y, Chen W, Hao Q, Ho C, Kennedy MK et al. Antibiotics for treatment of mild left-sided acute uncomplicated diverticulitis: meta-analysis of randomized trials. Br J Surg. 2023;110(3):373–4. 10.1093/bjs/znac433 36791230

[30] Jaung R, Nisbet S, Gosselink MP, Di Re A, Keane C, Lin A, et al. Antibiotics do not reduce length of hospital stay for uncomplicated diverticulitis in a pragmatic double-Blind randomized trial. Clin Gastroenterol Hepatol. 2021;19(3):503–10. e1. 32240832 10.1016/j.cgh.2020.03.049

[31] Au S, Aly EH. Treatment of uncomplicated acute diverticulitis without antibiotics: A systematic review and Meta-analysis. Dis Colon Rectum. 2019;62(12):1533–47. 30663999 10.1097/DCR.0000000000001330

[32] Rasheed W, Dweik A, Dharmarpandi G, Saeed A, Sohail AH, Shaikh MB, et al. Frailty in elderly patients with acute colonic diverticulitis is associated with worse in-hospital outcomes: a nationwide analysis. Ann Gastroenterol. 2024;37(5):552–8. 39238801 10.20524/aog.2024.0904 PMC11372536

[33] Gaber CE, Kinlaw AC, Edwards JK, Lund JL, Sturmer T, Peacock Hinton S, et al. Comparative effectiveness and harms of antibiotics for outpatient diverticulitis: two nationwide cohort studies. Ann Intern Med. 2021;174(6):737–46. 33617725 10.7326/M20-6315 PMC9035276

[34] Stephenson AL, Wu W, Cortes D, Rochon PA. Tendon injury and fluoroquinolone use: A systematic review. Drug Saf. 2013;36(9):709–21. 23888427 10.1007/s40264-013-0089-8

[35] Schug-Pass C, Geers P, Hugel O, Lippert H, Kockerling F. Prospective randomized trial comparing short-term antibiotic therapy versus standard therapy for acute uncomplicated sigmoid diverticulitis. Int J Colorectal Dis. 2010;25(6):751–9. 20140619 10.1007/s00384-010-0899-4

[36] Schneider EB, Singh A, Sung J, Hassid B, Selvarajah S, Fang SH, et al. Emergency department presentation, admission, and surgical intervention for colonic diverticulitis in the united States. Am J Surg. 2015;210(2):404–7. 26002192 10.1016/j.amjsurg.2014.12.050

[37] Lidsky ME, Thacker JK, Lagoo-Deenadayalan SA, Scarborough JE. Advanced age is an independent predictor for increased morbidity and mortality after emergent surgery for diverticulitis. Surgery. 2012;152(3):465–72. 22938905 10.1016/j.surg.2012.06.038

[38] Lidor AO, Schneider E, Segal J, Yu Q, Feinberg R, Wu AW. Elective surgery for diverticulitis is associated with high risk of intestinal diversion and hospital readmission in older adults. J Gastrointest Surg. 2010;14(12):1867–73. discussion 73– 4. 20878256 10.1007/s11605-010-1344-2

[39] Strate LL, Peery AF, Neumann I. American gastroenterological association Institute technical review on the management of acute diverticulitis. Gastroenterology. 2015;149(7):1950–76. e12. 26453776 10.1053/j.gastro.2015.10.001

[40] Stefansson T, Ekbom A, Sparen P, Pahlman L. Association between sigmoid diverticulitis and left-sided colon cancer: a nested, population-based, case control study. Scand J Gastroenterol. 2004;39(8):743–7. 15513359 10.1080/00365520410003272

[41] Meyer J, Orci LA, Combescure C, Balaphas A, Morel P, Buchs NC, et al. Risk of colorectal Cancer in patients with acute diverticulitis: A systematic review and Meta-analysis of observational studies. Clin Gastroenterol Hepatol. 2019;17(8):1448–e5617. 30056181 10.1016/j.cgh.2018.07.031

[42] Qaseem A, Etxeandia-Ikobaltzeta I, Lin JS, Fitterman N, Shamliyan T, Wilt TJ. Colonoscopy for diagnostic evaluation and interventions to prevent recurrence after acute Left-Sided colonic diverticulitis: A clinical guideline from the American college of physicians. Ann Intern Med. 2022;175(3):416–31. 35038270 10.7326/M21-2711

[43] Hall JF, Roberts PL, Ricciardi R, Read T, Scheirey C, Wald C, et al. Long-term follow-up after an initial episode of diverticulitis: what are the predictors of recurrence? Dis Colon Rectum. 2011;54(3):283–8. 21304297 10.1007/DCR.0b013e3182028576

[44] Liu PH, Cao Y, Keeley BR, Tam I, Wu K, Strate LL, et al. Adherence to a healthy lifestyle is associated with a lower risk of diverticulitis among men. Am J Gastroenterol. 2017;112(12):1868–76. 29112202 10.1038/ajg.2017.398 PMC5736501

[45] Humes DJ, Fleming KM, Spiller RC, West J. Concurrent drug use and the risk of perforated colonic diverticular disease: a population-based case-control study. Gut. 2011;60(2):219–24. 20940283 10.1136/gut.2010.217281

[46] Peery AF, Keku TO, Addamo C, McCoy AN, Martin CF, Galanko JA, et al. Colonic diverticula are not associated with mucosal inflammation or chronic Gastrointestinal symptoms. Clin Gastroenterol Hepatol. 2018;16(6):884–91. e1. 28603053 10.1016/j.cgh.2017.05.051 PMC5722710

[47] Lovell RM, Ford AC. Global prevalence of and risk factors for irritable bowel syndrome: a meta-analysis. Clin Gastroenterol Hepatol. 2012;10(7):712–21. e4. 22426087 10.1016/j.cgh.2012.02.029

[48] Gisbert JP, Chaparro M. Systematic review with meta-analysis: inflammatory bowel disease in the elderly. Aliment Pharmacol Ther. 2014;39(5):459–77. 24405149 10.1111/apt.12616

[49] Charpentier C, Salleron J, Savoye G, Fumery M, Merle V, Laberenne JE, et al. Natural history of elderly-onset inflammatory bowel disease: a population-based cohort study. Gut. 2014;63(3):423–32. 23408350 10.1136/gutjnl-2012-303864

[50] Brandt LJ, Feuerstadt P, Longstreth GF, Boley SJ. American college of G. ACG clinical guideline: epidemiology, risk factors, patterns of presentation, diagnosis, and management of colon ischemia (CI). Am J Gastroenterol. 2015;110(1):18–44. quiz 5. 25559486 10.1038/ajg.2014.395

[51] Montoro MA, Brandt LJ, Santolaria S, Gomollon F, Sanchez Puertolas B, Vera J, et al. Clinical patterns and outcomes of ischaemic colitis: results of the working group for the study of ischaemic colitis in Spain (CIE study). Scand J Gastroenterol. 2011;46(2):236–46. 20961178 10.3109/00365521.2010.525794

[52] Plastaras L, Vuitton L, Badet N, Koch S, Di Martino V, Delabrousse E. Acute colitis: differential diagnosis using multidetector CT. Clin Radiol. 2015;70(3):262–9. 25522900 10.1016/j.crad.2014.11.008

[53] Johansson N, Rosemar A, Angenete E. Risk of recurrence of sigmoid volvulus: a single-centre cohort study. Colorectal Dis. 2018;20(6):529–35. 29178415 10.1111/codi.13972

[54] Sugimoto T, Nagasue Y, Tanaka E, Yokomizo H. Comparison of the risk of appendiceal tumors in uncomplicated and complicated appendicitis. Surg Endosc. 2022;36(11):8107–11. 35449477 10.1007/s00464-022-09246-2

[55] Kawaguchi T, Araki Y, Kajiwara A, Nakao K, Sakakibara S, Sasaki Y, et al. A significant increase in the serum carbohydrate antigen 19– 9 level accompanied by acute cholecystitis and choledocholithiasis: A case report and review of the literature. Intern Med. 2024;63(12):1713–8. 37926542 10.2169/internalmedicine.2597-23 PMC11239259

[56] Loeb M, Bentley DW, Bradley S, Crossley K, Garibaldi R, Gantz N, et al. Development of minimum criteria for the initiation of antibiotics in residents of long-term-care facilities: results of a consensus conference. Infect Control Hosp Epidemiol. 2001;22(2):120–4. 11232875 10.1086/501875

[57] Rowe T, Towle V, Van Ness PH, Juthani-Mehta M. Lack of positive association between falls and bacteriuria plus Pyuria in older nursing home residents. J Am Geriatr Soc. 2013;61(4):653–4. 23581923 10.1111/jgs.12177 PMC3628627

[58] Juthani-Mehta M, Quagliarello V, Perrelli E, Towle V, Van Ness PH, Tinetti M. Clinical features to identify urinary tract infection in nursing home residents: a cohort study. J Am Geriatr Soc. 2009;57(6):963–70. 19490243 10.1111/j.1532-5415.2009.02227.x PMC2692075

[59] Strate LL, Ayanian JZ, Kotler G, Syngal S. Risk factors for mortality in lower intestinal bleeding. Clin Gastroenterol Hepatol. 2008;6(9):1004–10. quiz 955-. 18558513 10.1016/j.cgh.2008.03.021 PMC2643270

[60] Kaise M, Nagata N, Ishii N, Omori J, Goto O, Iwakiri K. Epidemiology of colonic diverticula and recent advances in the management of colonic diverticular bleeding. Dig Endosc. 2020;32(2):240–50. 31578767 10.1111/den.13547

[61] Nagata N, Niikura R, Aoki T, Shimbo T, Itoh T, Goda Y, et al. Increase in colonic diverticulosis and diverticular hemorrhage in an aging society: lessons from a 9-year colonoscopic study of 28,192 patients in Japan. Int J Colorectal Dis. 2014;29(3):379–85. 24317937 10.1007/s00384-013-1808-4

[62] Nagata N, Kobayashi K, Yamauchi A, Yamada A, Omori J, Ikeya T, et al. Identifying bleeding etiologies by endoscopy affected outcomes in 10,342 cases with hematochezia: CODE BLUE-J study. Am J Gastroenterol. 2021;116(11):2222–34. 34388140 10.14309/ajg.0000000000001413 PMC8560163

[63] Longstreth GF. Epidemiology and outcome of patients hospitalized with acute lower Gastrointestinal hemorrhage: a population-based study. Am J Gastroenterol. 1997;92(3):419–24. 9068461

[64] Lanas A, Garcia-Rodriguez LA, Polo-Tomas M, Ponce M, Alonso-Abreu I, Perez-Aisa MA, et al. Time trends and impact of upper and lower Gastrointestinal bleeding and perforation in clinical practice. Am J Gastroenterol. 2009;104(7):1633–41. 19574968 10.1038/ajg.2009.164

[65] Buttenschoen K, Buttenschoen DC, Odermath R, Beger HG. Diverticular disease-associated hemorrhage in the elderly. Langenbecks Arch Surg. 2001;386(1):8–16. 11405093 10.1007/s004230000198

[66] Mohammed Ilyas MI, Szilagy EJ. Management of diverticular bleeding: evaluation, stabilization, intervention, and recurrence of bleeding and indications for resection after control of bleeding. Clin Colon Rectal Surg. 2018;31(4):243–50. 29942215 10.1055/s-0037-1607963 PMC6014846

[67] Joaquim N, Caldeira P, Antunes AG, Eusebio M, Guerreiro H. Risk factors for severity and recurrence of colonic diverticular bleeding. Rev Esp Enferm Dig. 2017;109(1):3–9. 27925464 10.17235/reed.2016.4190/2015

[68] Kinjo K, Matsui T, Hisabe T, Ishihara H, Kojima T, Chuman K, et al. Risk factors for severity of colonic diverticular hemorrhage. Intest Res. 2018;16(3):458–66. 30090045 10.5217/ir.2018.16.3.458 PMC6077309

[69] Patel P, Siraw BB, Mehadi AY, Zaher EA, Ebrahim MA, Tafesse YT. Predictors of in-hospital outcomes for diverticular bleeding patients: a retrospective analysis of National Inpatient Sample data (2016–2020). Ann Gastroenterol. 10.20524/aog.2024.0896 PMC11226741 38974086

[70] Sengupta N, Feuerstein JD, Jairath V, Shergill AK, Strate LL, Wong RJ et al. Management of Patients With Acute Lower Gastrointestinal Bleeding: An Updated ACG Guideline. Am J Gastroenterol. 2023;118(2):208– 31. 10.14309/ajg.0000000000002130 36735555

[71] Obana T, Fujita N, Sugita R, Hirasawa D, Sugawara T, Harada Y, et al. Prospective evaluation of contrast-enhanced computed tomography for the detection of colonic diverticular bleeding. Dig Dis Sci. 2013;58(7):1985–90. 23504354 10.1007/s10620-013-2629-6

[72] Feuerstein JD, Ketwaroo G, Tewani SK, Cheesman A, Trivella J, Raptopoulos V, et al. Localizing acute lower Gastrointestinal hemorrhage: CT angiography versus tagged RBC scintigraphy. AJR Am J Roentgenol. 2016;207(3):578–84. 27303989 10.2214/AJR.15.15714

[73] Niikura R, Nagata N, Shimbo T, Aoki T, Yamada A, Hirata Y, et al. Natural history of bleeding risk in colonic diverticulosis patients: a long-term colonoscopy-based cohort study. Aliment Pharmacol Ther. 2015;41(9):888–94. 25715746 10.1111/apt.13148

[74] Cirocchi R, Grassi V, Cavaliere D, Renzi C, Tabola R, Poli G, et al. New trends in acute management of colonic diverticular bleeding: A systematic review. Med (Baltim). 2015;94(44):e1710. 10.1097/MD.0000000000001710 PMC4915869 26554768

[75] Niikura R, Nagata N, Yamada A, Honda T, Hasatani K, Ishii N, et al. Efficacy and safety of early vs elective colonoscopy for acute lower Gastrointestinal bleeding. Gastroenterology. 2020;158(1):168–75. e6. 31563627 10.1053/j.gastro.2019.09.010

[76] Sengupta N, Tapper EB, Feuerstein JD. Early versus delayed colonoscopy in hospitalized patients with lower Gastrointestinal bleeding: A Meta-Analysis. J Clin Gastroenterol. 2017;51(4):352–9. 27466163 10.1097/MCG.0000000000000602

[77] Kumar NL, Cohen AJ, Nayor J, Claggett BL, Saltzman JR. Timing of upper endoscopy influences outcomes in patients with acute nonvariceal upper GI bleeding. Gastrointest Endosc. 2017;85(5):945–52. e1. 27693643 10.1016/j.gie.2016.09.029

[78] Oakland K, Jairath V, Uberoi R, Guy R, Ayaru L, Mortensen N, et al. Derivation and validation of a novel risk score for safe discharge after acute lower Gastrointestinal bleeding: a modelling study. Lancet Gastroenterol Hepatol. 2017;2(9):635–43. 28651935 10.1016/S2468-1253(17)30150-4

[79] Villanueva C, Colomo A, Bosch A, Concepcion M, Hernandez-Gea V, Aracil C, et al. Transfusion strategies for acute upper Gastrointestinal bleeding. N Engl J Med. 2013;368(1):11–21. 23281973 10.1056/NEJMoa1211801

[80] Nightingale N, Zou G, Murphy MF, Jairath V. Revisiting triggers: optimal thresholds for transfusion in Gastrointestinal bleeding May be higher than restrictive guidelines. Clin Gastroenterol Hepatol. 2023;21(7):1955–6. 35840062 10.1016/j.cgh.2022.06.006

[81] Zakko L, Rustagi T, Douglas M, Laine L. No benefit from platelet transfusion for Gastrointestinal bleeding in patients taking antiplatelet agents. Clin Gastroenterol Hepatol. 2017;15(1):46–52. 27464591 10.1016/j.cgh.2016.07.017

[82] Chan FK, Leung Ki EL, Wong GL, Ching JY, Tse YK, Au KW, et al. Risks of bleeding recurrence and cardiovascular events with continued aspirin use after lower Gastrointestinal hemorrhage. Gastroenterology. 2016;151(2):271–7. 27130815 10.1053/j.gastro.2016.04.013

[83] Wolf AT, Wasan SK, Saltzman JR. Impact of anticoagulation on rebleeding following endoscopic therapy for nonvariceal upper Gastrointestinal hemorrhage. Am J Gastroenterol. 2007;102(2):290–6. 17100959 10.1111/j.1572-0241.2006.00969.x

[84] Goldstein JN, Refaai MA, Milling TJ Jr., Lewis B, Goldberg-Alberts R, Hug BA, et al. Four-factor prothrombin complex concentrate versus plasma for rapid vitamin K antagonist reversal in patients needing urgent surgical or invasive interventions: a phase 3b, open-label, non-inferiority, randomised trial. Lancet. 2015;385(9982):2077–87. 25728933 10.1016/S0140-6736(14)61685-8 PMC6633921

[85] Chai-Adisaksopha C, Hillis C, Siegal DM, Movilla R, Heddle N, Iorio A, et al. Prothrombin complex concentrates versus fresh frozen plasma for warfarin reversal. A systematic review and meta-analysis. Thromb Haemost. 2016;116(5):879–90. 27488143 10.1160/TH16-04-0266

[86] Smith SR, Murray D, Pockney PG, Bendinelli C, Draganic BD, Carroll R. Tranexamic acid for lower GI hemorrhage: A randomized Placebo-Controlled clinical trial. Dis Colon Rectum. 2018;61(1):99–106. 29215478 10.1097/DCR.0000000000000943

[87] Collaborators H-IT. Effects of a high-dose 24-h infusion of Tranexamic acid on death and thromboembolic events in patients with acute Gastrointestinal bleeding (HALT-IT): an international randomised, double-blind, placebo-controlled trial. Lancet. 2020;395(10241):1927–36. 32563378 10.1016/S0140-6736(20)30848-5 PMC7306161

[88] Bruce G, Erskine B. Analysis of time delay between computed tomography and digital Subtraction angiography on the technical success of interventional embolisation for treatment of lower Gastrointestinal bleeding. J Med Radiat Sci. 2020;67(1):64–71. 31886625 10.1002/jmrs.373 PMC7063255

[89] Gobinet-Suguro M, Nagata N, Kobayashi K, Yamauchi A, Yamada A, Omori J, et al. Treatment strategies for reducing early and late recurrence of colonic diverticular bleeding based on stigmata of recent hemorrhage: a large multicenter study. Gastrointest Endosc. 2022;95(6):1210–22. e12. 34979112 10.1016/j.gie.2021.12.023

[90] Vajravelu RK, Mamtani R, Scott FI, Waxman A, Lewis JD, Incidence. Risk factors, and clinical effects of recurrent diverticular hemorrhage: A large cohort study. Gastroenterology. 2018;155(5):1416–27. 30056095 10.1053/j.gastro.2018.07.026 PMC6219900

[91] Strate LL, Liu YL, Huang ES, Giovannucci EL, Chan AT. Use of aspirin or nonsteroidal anti-inflammatory drugs increases risk for diverticulitis and diverticular bleeding. Gastroenterology. 2011;140(5):1427–33. 21320500 10.1053/j.gastro.2011.02.004 PMC3081980

[92] Nagata N, Niikura R, Aoki T, Moriyasu S, Sakurai T, Shimbo T, et al. Risk factors for adverse in-hospital outcomes in acute colonic diverticular hemorrhage. World J Gastroenterol. 2015;21(37):10697–703. 26457031 10.3748/wjg.v21.i37.10697 PMC4588093

[93] Nagata N, Niikura R, Aoki T, Shimbo T, Sekine K, Okubo H, et al. Impact of discontinuing non-steroidal antiinflammatory drugs on long-term recurrence in colonic diverticular bleeding. World J Gastroenterol. 2015;21(4):1292–8. 25632204 10.3748/wjg.v21.i4.1292 PMC4306175

[94] Kirita K, Kodaka Y, Shibata Y, Ueki N, Agawa S, Yamawaki H, et al. Impact of clinical characteristics of colonic diverticular bleeding in extremely elderly patients treated with direct oral anti-coagulant drugs: a retrospective multi-center study. J Clin Biochem Nutr. 2021;69(2):222–8. 34616113 10.3164/jcbn.20-140 PMC8482383

[95] Jaruvongvanich V, Prasitlumkum N, Assavapongpaiboon B, Suchartlikitwong S, Sanguankeo A, Upala S. Risk factors for delayed colonic post-polypectomy bleeding: a systematic review and meta-analysis. Int J Colorectal Dis. 2017;32(10):1399–406. 28779355 10.1007/s00384-017-2870-0

[96] Johanson JF, Sonnenberg A. The prevalence of hemorrhoids and chronic constipation. An epidemiologic study. Gastroenterology. 1990;98(2):380–6. 2295392 10.1016/0016-5085(90)90828-o

[97] Ahmad H, Jannat H, Khan U, Ahmad N. Stercoral colitis: A diagnostic challenge and therapeutic approach in an elderly patient with chronic constipation. Cureus. 2023;15(5):e39179. 37378172 10.7759/cureus.39179 PMC10292177

[98] Notsu T, Adachi K, Mishiro T, Kishi K, Ishimura N, Ishihara S. Prevalence of angiodysplasia detected in upper Gastrointestinal endoscopic examinations. Cureus. 2021;13(4):e14353. 33972910 10.7759/cureus.14353 PMC8105191

[99] Tariq T, Karabon P, Irfan FB, Goyal S, Mayeda MM, Parsons A, et al. Secondary angiodysplasia-associated Gastrointestinal bleeding in end-stage renal disease: results from the nationwide inpatient sample. World J Gastrointest Endosc. 2019;11(10):504–14. 31798771 10.4253/wjge.v11.i10.504 PMC6885446

[100] Goltstein L, Rooijakkers MJP, Hoeks M, Li WWL, van Wely MH, Rodwell L, et al. Effectiveness of aortic valve replacement in Heyde syndrome: a meta-analysis. Eur Heart J. 2023;44(33):3168–77. 37555393 10.1093/eurheartj/ehad340 PMC10471563

